# Dolomite genesis in bioturbated marine zones of an early-middle Miocene coastal mud volcano outcrop (Kuwait)

**DOI:** 10.1038/s41598-021-85978-w

**Published:** 2021-03-23

**Authors:** Ammar Alibrahim, Michael J. Duane, Maria Dittrich

**Affiliations:** 1 Department of Physical and Environmental Sciences, University of Toronto Scarborough, 1265 Military Trail, Toronto, ON M1C 1A4 Canada; 2 Department of Earth and Environmental Sciences, Kuwait University, P.O. Box 5969, 13060 Safat, Kuwait

**Keywords:** Biogeochemistry, Environmental sciences, Solid Earth sciences

## Abstract

The origin of spheroidal dolomitized burrow from Al-Subiya sabkha in Kuwait was previously described as enigmatic as no evidence of precursor calcium carbonate was found in the siliciclastic sediment. An assumption for the genesis of spheroidal dolomite from the same area was attributed to hydrocarbon seepage but no evidence was provided. Here, we investigated a recently discovered early-middle Miocene coastal mud volcano outcrop in Al-Subiya sabkha where dolomitized burrows and spheroidal dolomite are found in bioturbated marine zones, and associated with traces of salt. Conversely, the continental zone lacks bioturbation features, dolomite and traces of salt, which together contrast with bioturbated rich marine zones. Geochemical signatures of Rare Earth Elements + Yttrium show a true positive Ce anomaly (Ce/Ce* > 1.2) and positive Eu/Eu* anomaly of spheroidal dolomite indicating strictly anoxic conditions, and sulphate reduction to sulphide, respectively. Our results are suggestive of a relationship between dolomite formation and interdependent events of hydrocarbon seepage, flux of hypersaline seawater, bioturbation, and fluid flow in the marine zones of the mud volcano. The bioturbation activity of crustaceans introduced channels/burrows in the sediment–water interface allowing for the mixing of seeped pressurized hydrocarbon-charged fluids, and evaporitic seawater. In the irrigated channels/burrows, the seeped pressurized hydrocarbon-charged fluids were oxidized via microbial consortia of methanotrophic archaea and sulphate-reducing bacteria resulting in elevated alkalinity and saturation index with respect to dolomite, thus providing the preferential geochemical microenvironment for dolomite precipitation in the bioturbated sediment.

## Introduction

The argument over the origin of dolomite remains ongoing and is referred to as “the dolomite problem”^1,2^. The essence of the dolomite problem is based on the disparity of dolomite over the geologic time scale where massive dolomite was forming in ancient sedimentary rocks but decreased in modern depositional environments. In addition, there is a persistent inability to precipitate dolomite abiotically in laboratory experiments simulating the Earth’s surface conditions^3,4^. Attempts to solve the multifaceted “dolomite problem” have shed some light on important factors controlling dolomite formation including high salinity^5,6^, dissolution–precipitation reaction of intermediate phases^7^, redox conditions^8^, destabilizing the hydration shell of Mg^2+^ ^9^, high concentration of dissolved Mg^2+^and Ca^2+^ ions^10^, microbial sulphate reduction^11,12^, and high density of carboxylic groups^13^ as nucleation sites within microbial biofilm, and cells^14,15^. Although numerous factors were identified, the origin of dolomite remains an outstanding problem.

Dolomite formation in the sabkha environment was essentially related to active evaporation of seawater^2^. In the coastal area of the Arabian Gulf, the formation of modern dolomite in the evaporitic shallow marine sabkha was found to be coupled with microbial extracellular polymeric substances (EPS)^16–20^ while the diagenetic events were reported to be consequences of sabkha flood recharge/reflux, and concentrated seawater^21–24^. In the northern region of the Arabian Gulf (Kuwait), an exceptional spheroidal dolomitized burrow was found on the surface of modern clastic tidal-flat sabkha complex with neither evidence of EPS involvement nor diagenetic features^25^. Interestingly, spheroidal dolomite cements from exposed Eocene to Quaternary rocks in north Kuwait were assumed to be related to hydrocarbon seepage. The preferential morphology of spheroids over the rhombic dolomite was proposed to be related to the oxidation of seeping hydrocarbon-charged groundwater which produces carbon dioxide (CO_2_) bubbles that act as the nuclei for spheroidal dolomite to crystallize by means of microbial inducement^26^. Although more than 30 years have passed since the discovery of the enigmatic spheroidal dolomitized burrow as well as the proposed hydrocarbon-related dolomite genesis model in north Kuwait, the mechanism of dolomite formation remains unsubstantiated and the geochemical conditions remain unclear. Therefore, it is worthy to highlight the environmental conditions of Al-Subiya sabkha where hydrocarbon seepage and hypersaline sabkha of Miocene age had interacted in favour of dolomite formation in this exceptional sabkha.

In the present work, we propose a novel formation mechanism for the previously described burrow and spheroidal dolomite^25,26^. Our proposed mechanism includes an interdependent sequence of events comprising hydrocarbon seepage, flux of hypersaline seawater from the shallow sabkha, and bioturbation introduced by crustaceans. Our investigation has focused on a recently discovered coastal mud volcano outcrop of early-middle Miocene age, with associated prolific Decapoda burrows^27,28^. The sediment-modifying ability of Decapoda by burrowing and fluids transporting between various depths of the seafloor is well known from the fossil record, and in the modern context^29^. *Ophiomorpha*^30^ and *Thalassinoides*^31^ are the most abundant ichnogenera related to prolific cavities in the studied mud volcano and are well described by *Hyžný *et al*.* 2018^28^. Lime mudstone with bioturbation structures exhibits high permeability and porosity allowing the discharge of fluids both through the burrows^32^ and vents ^27^. In hydrocarbon seepage sites, anaerobic oxidation of the seeped methane within the burrows can be mediated by consortia of methanotrophic archaea and sulphate-reducing bacteria^33,34^. The oxidation of methane increases alkalinity^35^ with a consequent selective-dolomitization of the burrows^25,36^. The source of magnesium for dolomitization is assumed to be the evaporitic seawater from the shallow sabkha as bio-irrigation enhances the mixing of overlying water with solutes from the sediment^37^, and fills the burrows via advective transport^38^.

This paper presents a re-evaluation of the genesis of dolomitized burrows and spheroidal dolomite in Al-Subiya sabkha based on the interpretation of field observation, mineralogic, petrographic, and geochemical analyses. This paper attributes the disparity of dolomite abundance in contrasting zones of the mud volcano to hypersaline seawater and the activity of burrowing crustaceans. The marine zones where dolomite was found exhibit marine trace fossils and evaporites that were lacking in the fine-grained continental zone.

## Site description and sampling

**Figure 1:**
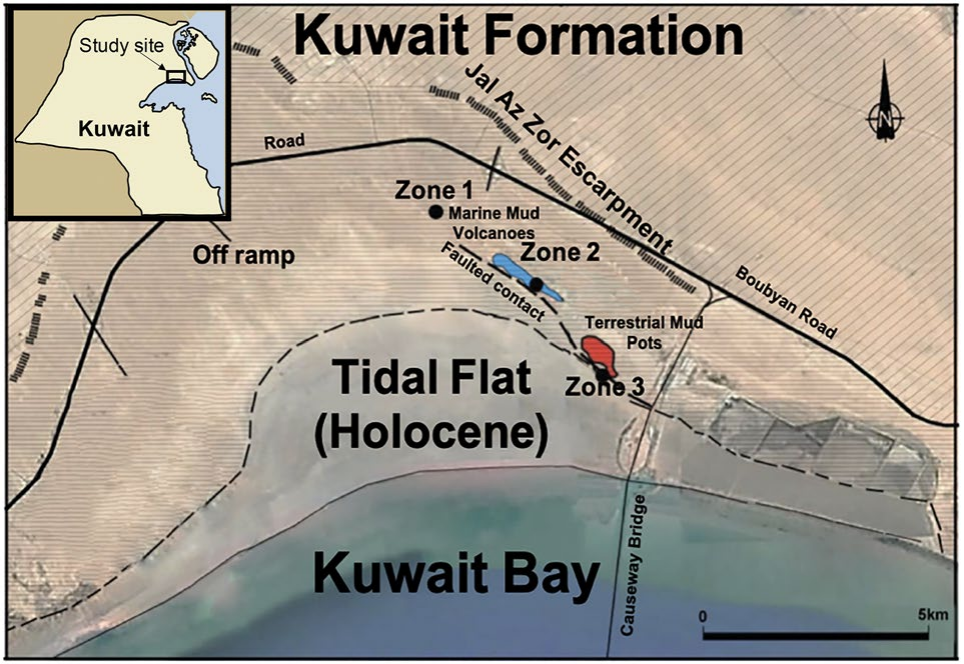
Aerial image from Google Maps showing Al-Subiya area in northern Kuwait. Sampling focused on 3 different lower Miocene outcrops, separated by Holocene remnant channels. Kuwait map (top-left) and the outline were created using Adobe Photoshop version 22.0.1 (https://www.adobe.com/).

**Figure 2:**
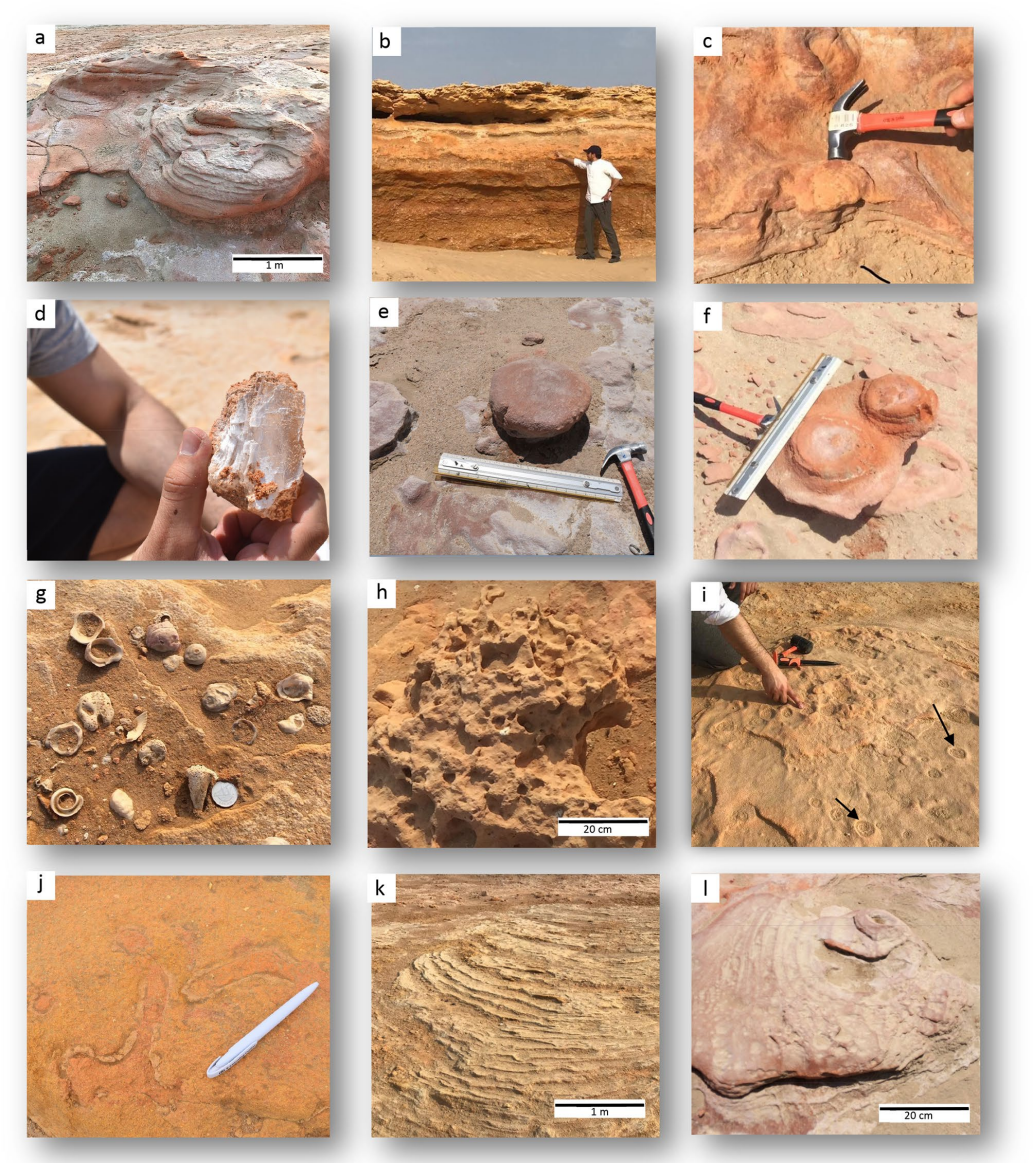
Features of the Miocene mud volcano field. (**a**) Large crater with concentrically developed banding; (**b**) Exposed Miocene strata showing 3 layers of; caliche in top, micro breccia in middle, and mega breccia at bottom; (**c**) Pipe-like structure with sandy matrix (MV1); (**d**) Evaporite megacrystic gypsum; (**e**) Concretion mound of (biogenic?) carbonate (MV2); (**f**) Lithified vents with regular annular radial pattern (MV3); (**g**) Original cover of Recent gastropod and bivalve shells on top of mud volcano outcrop; (**h**) Three-dimensional bioturbation network of *Thalassinoides* in carbonate rich breccia; (**i**) Surficial pockmarks pointed out by the arrows and are indicative of gas venting; (**j**) *Ophiomorpha* showing thick pelletoidal walls in evaporitic sandy matrix (**k**) Example of seismite, laminated sands and evaporites showing pseudo-bedding structures; (**l**) Weathered mixed breccia, concentric mound cemented by authigenic carbonate.

**Figure 3:**
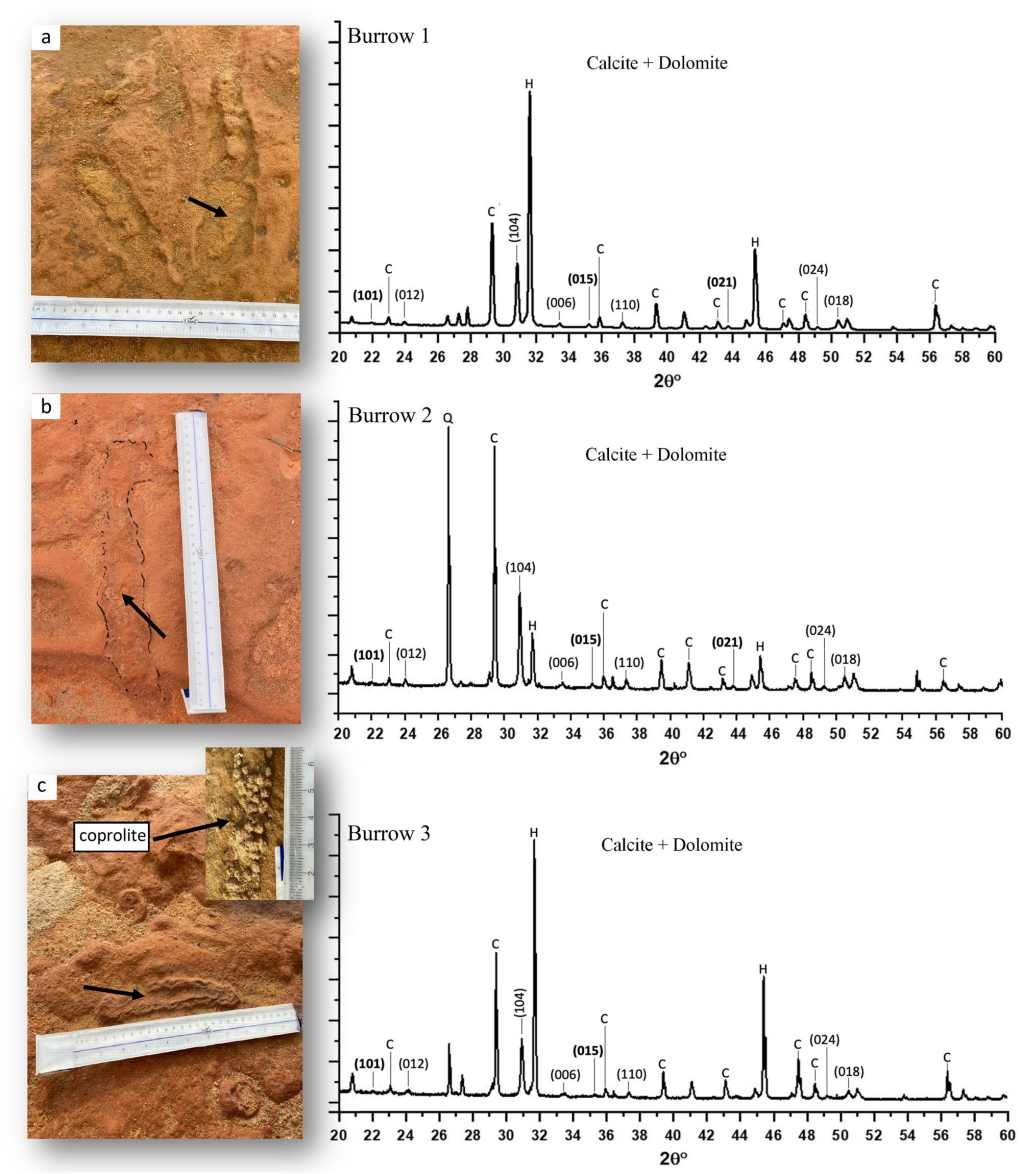
Strongly bioturbated zone in the marine section of the mud volcano complex adjacent to MV1 and MV2 samples (all images are top-view). (**a**) branched *Ophiomorpha* burrow (Decapoda). (**b**) Preserved interior burrow. (**c**) Unbranched *Ophiomorpha* burrow with pelleted walls, evacuated interior, and fossilized crustacean feces (coprolite). Compare this morphology to dolomitizing burrows with *Gunatilaka* et al. 1987. Powder XRD patterns (Cu_Kα_) of the three burrows show high concentration of halite, dolomite, and calcite. Dolomite peaks were labeled based on miller indices and ordering reflections are in bold. The letters Q, C, and H correspond to quartz, calcite, and halite, respectively.

The study area is located in the northeast arch of Kuwait bay within the post Eocene Kuwait Formation, a representative strata of the traditional tripartite subdivision of Ghar, Lower Fars, and Dibdibba formations^39,40^. The subsurface formation of this area was recently reinterpreted in relation to prolonged tectonic compression and strike-slip tectonics. Thin-skinned tectonics by bedding parallel slippage of the Dammam and Rus formations from the south of Kuwait caused northward transgression of the Jal-Azour escarpment and opened up splay faults for subsurface fluids and gases to escape^41^. Within the basal part of Kuwait Formation, an early-middle Miocene mud volcano outcrop was the focus of our study. The current exposures of mud volcano features after seawater regression demonstrate pseudo-bioherm formations that display cratered elliptical-shapes indicative of seismicity and the consequent ascending fluids from subsurface plumbing systems^27^. Geometries of chimney-like structures were described as evaporitic mud pipes and canonical mounds that were formed due to extensive gas venting^27^. The burrows are restricted to the marine zones while the pockmarks are ubiquitous throughout the complex. Rocks were sampled from three different zones, two marine and one continental, separated by a remnant tidal channel. The coordinates for each sampling locations are (29°38′07.1"N 47°59′43.4"E) for site 1, (29°36′56.2"N 48°00′57.6"E) for site 2, and (29°36′07.0"N 48°01′53.7"E) for site 3. The rocks are labelled as MV1, MV2, and MV3 corresponding to zones 1, 2, and 3, respectively. Figure 1 shows the location of the mud volcano outcrop and the sampling sites. An overview of the features of the investigated area and the sampled rocks is displayed in Fig. 2. Figure 3 shows burrows from the marine zones and these burrows are comparable in size, shape, and mineralogy to published data on the same site ^25,28^. The detailed description of the biota and the associated ichnofabric has been previously described^28^.

## Methods and analytical techniques

### Powder X-ray diffraction (XRD)

The mineralogy of the burrows and rocks was determined using an X-ray diffractometer. The cores of the burrows were scooped out using a chisel and hammer and placed in sterile bags. MV1, MV2, and MV3 rocks were sampled and a representative fragment of each rock was cut using a wire diamond saw. All samples were pulverized into a fine powder using pestle and mortar pre-washed with isopropanol. The powdered samples were then loaded into the sample holder and analyzed on D8 Advance XRD (BRUKER, USA) for the burrows and a PHILIPS Analytical X-ray B.V. (PHILIPS, Netherlands) for the rocks. The detailed scanning settings were as follows: scan type Gonio with continuous mode, 2 $\theta$ scanning starting from 20° to 60° with Cu-Kα radiation, and step size of 0.02° with scan speed of 0.020^°^/s. X’Pert Quantify software was used for scanning, HighScorePlus software was used for peak analysis, and comparisons were made with the database of the International Center for Diffraction Data (ICDD). Spectra were plotted using Origin 2018 software (OriginLab Corporation, MA, USA).

### Electron probe microanalysis (EPMA)

EPMA was performed for the rocks to identify the quantitative compositional mapping of mineral phases and dolomite stoichiometry. Small fragments of the rocks were embedded in a 1-inch diameter mold and immersed in a mixture of epoxy. The epoxy mixture was prepared by adding 3 g of Epoxy Hardener (EpoThin 2) to 6 g of Epoxy Resin (EpoThin 2) then mixed and left overnight at room temperature to dry. The dried samples were ground and polished automatically (MetaServ 250 Grinding/Polishing—Buehler) in 3 steps using different grit size sandpaper of 240, 600, and 1,200, respectively rotating for 10 min/step. Further fine polishing was performed using different sizes of oil based monocrystalline diamond suspensions of 3 µm and 1 µm rotating for 15 min and 10 min, respectively. The speed of all rotating polishing disks was set at 200 rpm. After each polishing step with the diamond suspension, the samples were immersed in isopropyl alcohol and sonicated for 2 min to remove the excess suspension. Finally, the samples were coated with 15 nm carbon (Edwards Coating System—E306A) before analysis. The analysis was performed using the JEOL JXA-8230 EPMA (JEOL, Japan) with accelerating voltage at 15 kV, beam current at 10 nA, and focused beam width maintained at 10 µm. The counting time on each peak varied between 10 to 60 s and for each of the background from 5 to 30 s. Mineral compositional mapping was calculated based on internal standards of elemental composition; dolomite was further verified by using a secondary standard (806 dolomite sx2). Dolomite stoichiometry was determined by quantifying elements in the single point analyses. The standards for quantifying elements in single point for dolomite are (803 dolomite PS-89/6) for Mg and Ca, (226 haematsx1) for Fe, and (824 kutnahorite PS89/6) for Mn. Elements were quantified in oxide forms and CO_3_ was calculated from the dolomite standard. PC-EPMA software v1.15.0.0 (JEOL, Japan) was used for imaging and spectral analysis.

### Scanning electron microscopy with energy dispersive spectroscopy (SEM–EDS)

SEM–EDS technique characterizes the morphology and texture of rock grains in sub-micrometer spatial resolution with semi-quantitative elemental microanalysis. Small grains from the rocks were transferred on a double carbon tape on an aluminium stub and coated with 10 nm of osmium tetroxide (OsO_4_) (Filgen, Japan) before imaging. Samples were examined using a Hitachi S530 SEM (HITACHI, Japan) with emission current adjusted to 60 µA and voltage to 20 kV. Quartz PCI version 8 image management system (QUARTZ, Canada) was used for image acquisition. Elemental analysis of rock grains by EDS was performed using a JEOL JSM-6610LV SEM (JEOL, Japan) and equipped with the X-Max EDS system (Oxford Instruments, High Wycombe, UK). INCA software (Oxford Instruments, High Wycombe, UK) was used for data acquisition and processing.

### Cathodoluminescence microscopy (SEM-CL)

SEM-CL was used to inspect dolomite concentric radial fabric from thin sections of the rocks. A JEOL JSM-6610LV SEM (JEOL, Japan) was used with a cathodoluminescence detector (Gatan—MiniCL). Thin sections were prepared from the Epoxy-embedded samples used for EPMA. The embedded samples were glued on frosted petrographic glass slides. The bulk portion of the sample was cut by a diamond saw (IsoMet 4000) to obtain a thin layer on the slide. Grinding of the samples was processed using a PetroThin Thin Section Machine to attain a flat surface on the slide. Slides were then placed in a lapping and polishing machine (LOGITECH, LP30) using silicon carbide of 600 grit size (15 µm grain size) in order to obtain a sample thickness of 30 µm. An automated polishing system with an oil-based diamond suspension was applied in 3 steps: at 9 µm, 3 µm, and 1 µm consecutively, each step consisting of 5 min intervals (MetaServ 250 Grinding/Polishing—Buehler) at a speed of 200 rpm.

### Laser ablation inductively coupled plasma mass spectrometry (LA-ICPMS)

The concentration of the Rare Earth Elements, Yttrium (Y), Uranium (U), and Thorium (Th) were determined in 10 sub-samples of the rocks. Samples were prepared following the same protocol for EPMA but with two additional polishing steps of 0.3 mm Alumina and 0.06 mm colloidal silica using a polishing cloth. A pre-quantification of major elements (Ca, Mg, and Si) was initially performed by EPMA in order to correct LA-ICPMS primary results. LA-ICPMS analysis was performed using UP-213 laser ablation system coupled with VG PQ ExCell inductively coupled plasma mass spectrometry (ICPMS) system. Helium was used to transfer the ablated samples to ICPMS system and Argon was used to cool the torch. The settings of the laser were as follows: spot size ranged from 55 to 100 mm, scan speed was 20 mm/s, repetition rate was 10 Hz, and the laser percentage output power was 65%. The energy of the laser beam ranged from 0.10 to 0.40 mJ and the energy fluence rate was 4.19 J/cm^2^ to 6.51 J/cm^2^. The machine was tuned for maximum sensitivity and low oxide production to avoid the interference of oxides with the results. The oxides were standardized to be less than 2% by monitoring Th^232^/ThO^+248^ to be less than 2% and U^238^/Th^232^ ~ 1%. Before starting the analysis, standardization of the values was performed based on the standard NIST610 synthetic glass for correcting data drift. Two lines were ablated on the standard glass before analysis and two at the end of the analysis. The values of the measured elements were corrected after EPMA quantification and normalized to shale average of Post Australian Archean Shale (PAAS)^42^. GraphPad Prism software version 8 (GraphPad, CA, USA) was used for graphical representations.

## Results

### Mineralogical composition of authigenic carbonates

**Figure 4:**
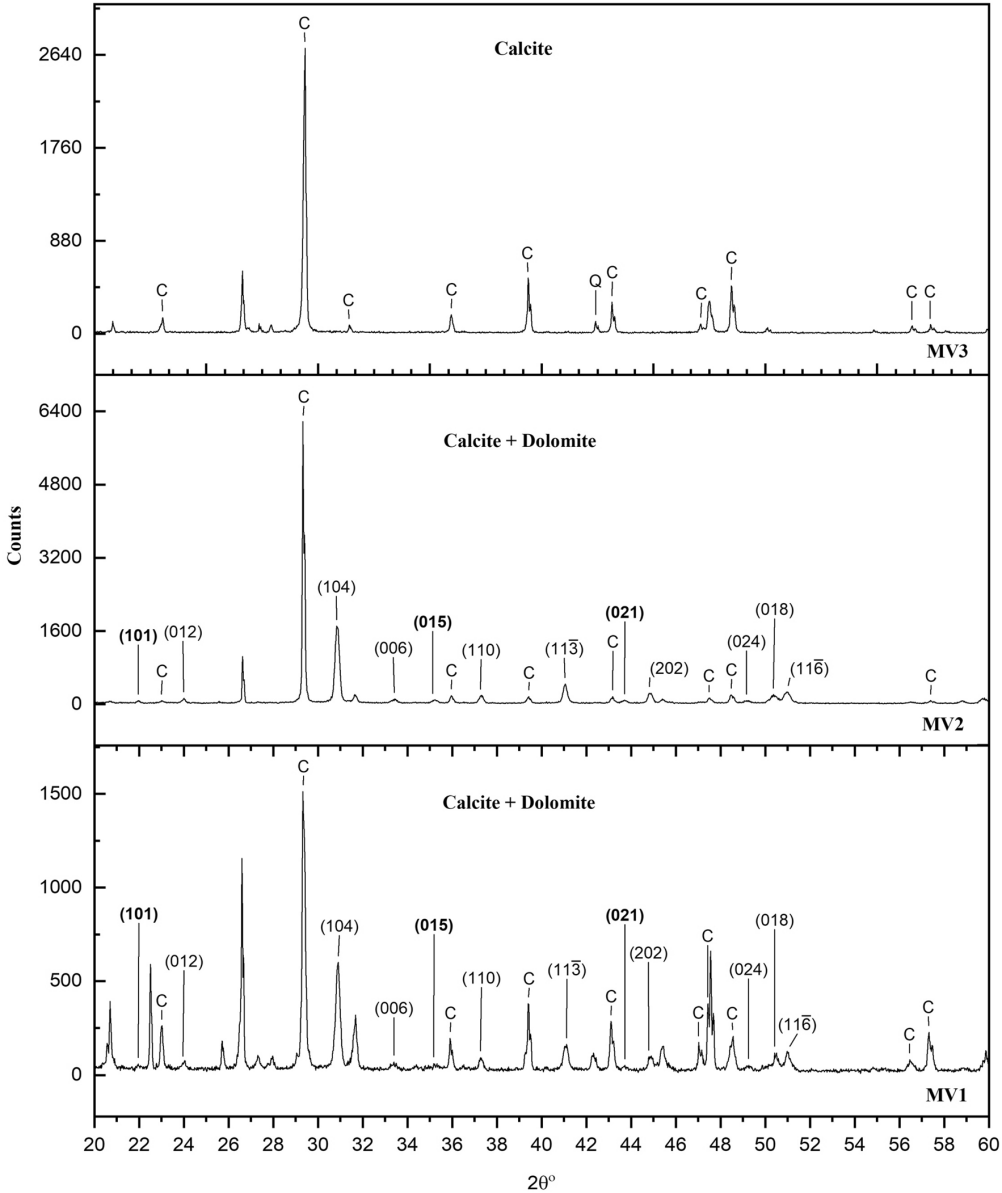
Powder XRD patterns (Cu_Kα_) of the three rock samples. Calcite and dolomite dominate MV1 and MV2 but only calcite dominates MV3. The non-superimposed major (104) peak of dolomite in MV1 and MV2 shows slightly different 2θ resulting from difference in calcium enrichment.

The mineralogic contents of the burrows, MV1, and MV2 are mainly calcite, halite, and dolomite while MV3 is dominated by calcite with the absence of halite and dolomite. The burrows from marine sites adjacent to MV1 and MV2 samples have a similar XRD pattern (Fig. 3) in comparison to MV1 and MV2 rocks (Fig. 4) but the peaks display varying intensities. Dolomite is discerned from other phases of CaMg(CO_3_)_2_ from the space group symmetry *R* $\stackrel{-}{3}$ resulting from the present ordering reflections of (101), (015), and (021), which are the distinguishable criteria of ordered dolomite^43^. Dolomite in the burrows, MV1, and MV2 matches different dolomite standards varying in stoichiometry and ordering degree. Dolomite stoichiometry ranges from Ca_1.07_Mg_0.93_(CO_3_)_2_ to stoichiometric CaMg(CO_3_)_2_ and the ordering degree ranges from 0.44 to 0.66 calculated from the intensity ratio of (105) and (110) reflections: *I*_(105)_/*I*_(110)_^44^. The attenuation of ordering indicates Ca^2+^ ions are occupying Mg^2+^ positions and vice versa ^43^. The peaks labeled with the letter (C) denote the mineral calcite, and dolomite peaks are labeled with the Miller indices where the ordering reflections are in bold (Figs. 3, 4). The major peaks for quartz correspond to 2 $\theta$ = 20.81 and 26.6, for halite correspond to 2 $\theta$ = 31.68 and 45.41, and minor silicates clay mineral correspond to 2 $\theta$ = 27.9.

**Figure 5:**
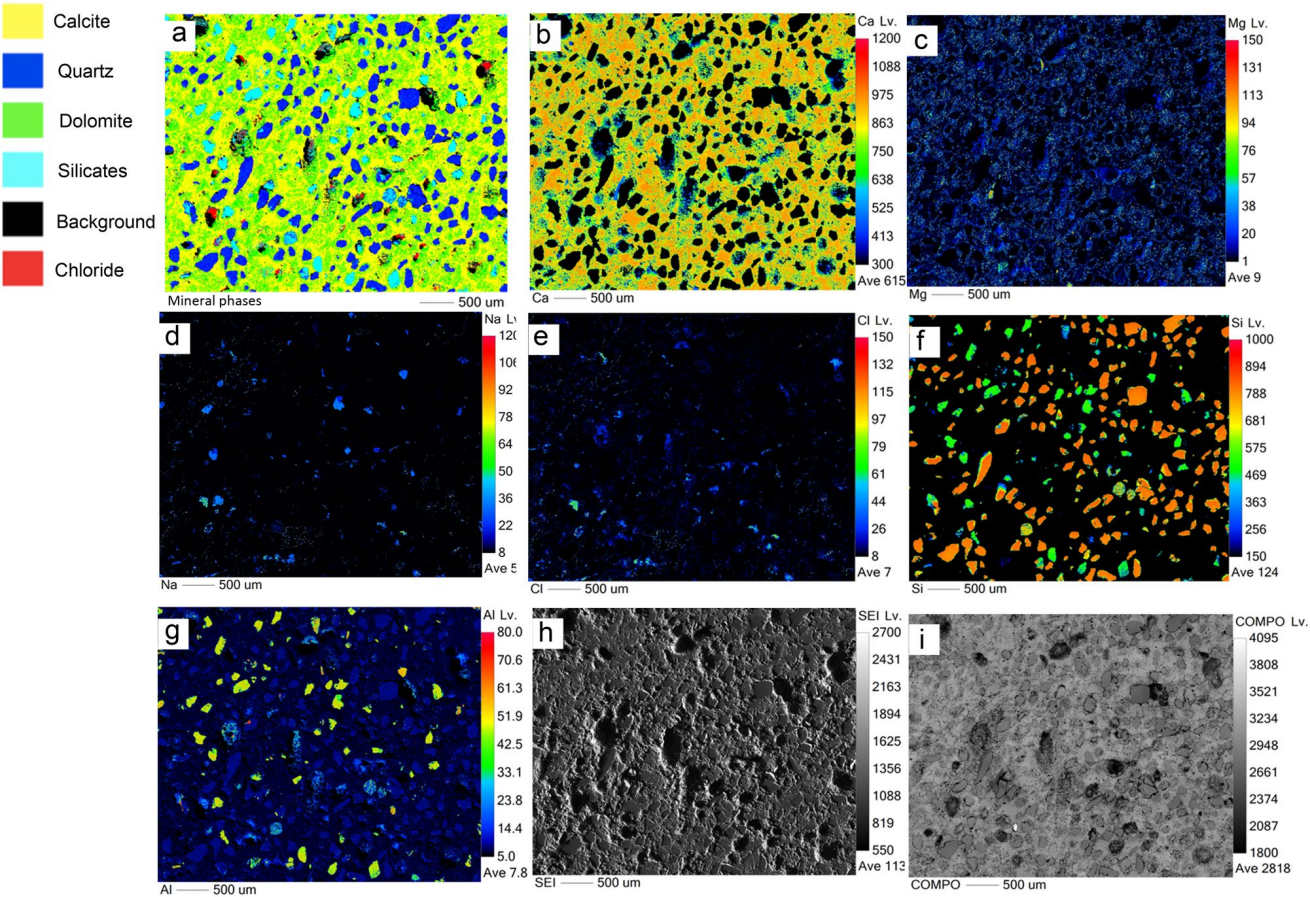
EPMA results of MV1 showing: (**a**) mineralogic maps, elemental maps of (**b**) Ca, (**c**) Mg, (**d**) Na, (**e**) Cl, (**f**) Si, and (**g**) Al, (**h**) secondary electron image, and (**i**) backscattered image. Dolomite microcrystals show a mesh-like structure that possibly indicating dissolution of susceptible dolomite. The absence of fossils suggests dolomitization in the upper tidal flat environment.

**Figure 6:**
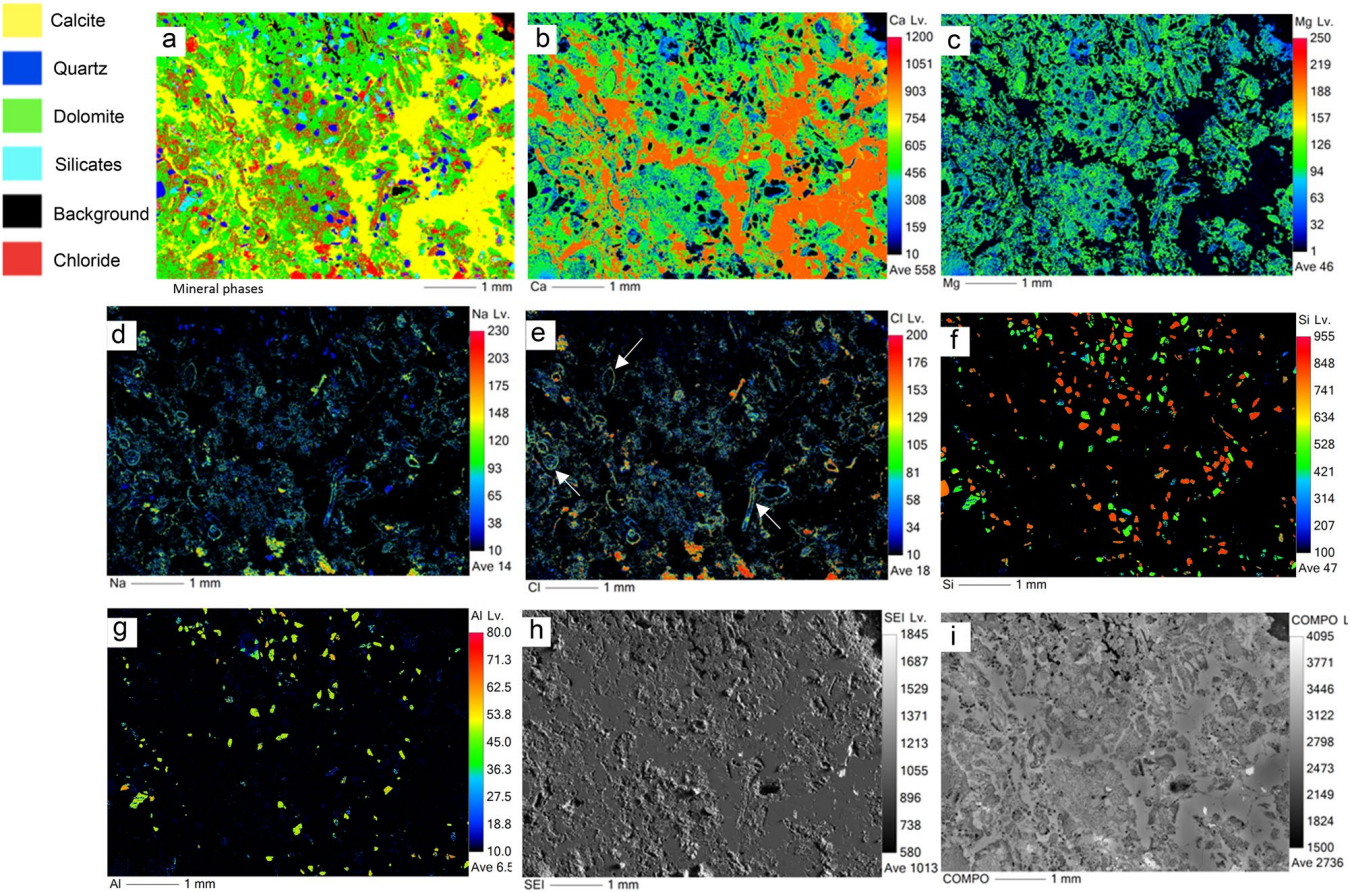
EPMA results of MV2 showing: (**a**) mineralogic maps, elemental maps of (**b**) Ca, (**c**) Mg, (**d**) Na, (**e**) Cl, (**f**) Si, and (**g**) Al, (**h**) secondary electron image, and (**i**) backscattered image. Arrows in panel (**e**) point microfossils, likely foraminifera. Some foraminifera in the coastal mud volcano were identified by Hyžný et al. (2018) such as *Elphidiella* sp.; *Elphidium* cf. *macellum*; *Praeorbulina glomerosa*; *Quinqueloculina seminula*; *Quinqueloculina badenensis*; and *Spiroloculina tenuis.*

**Figure 7:**
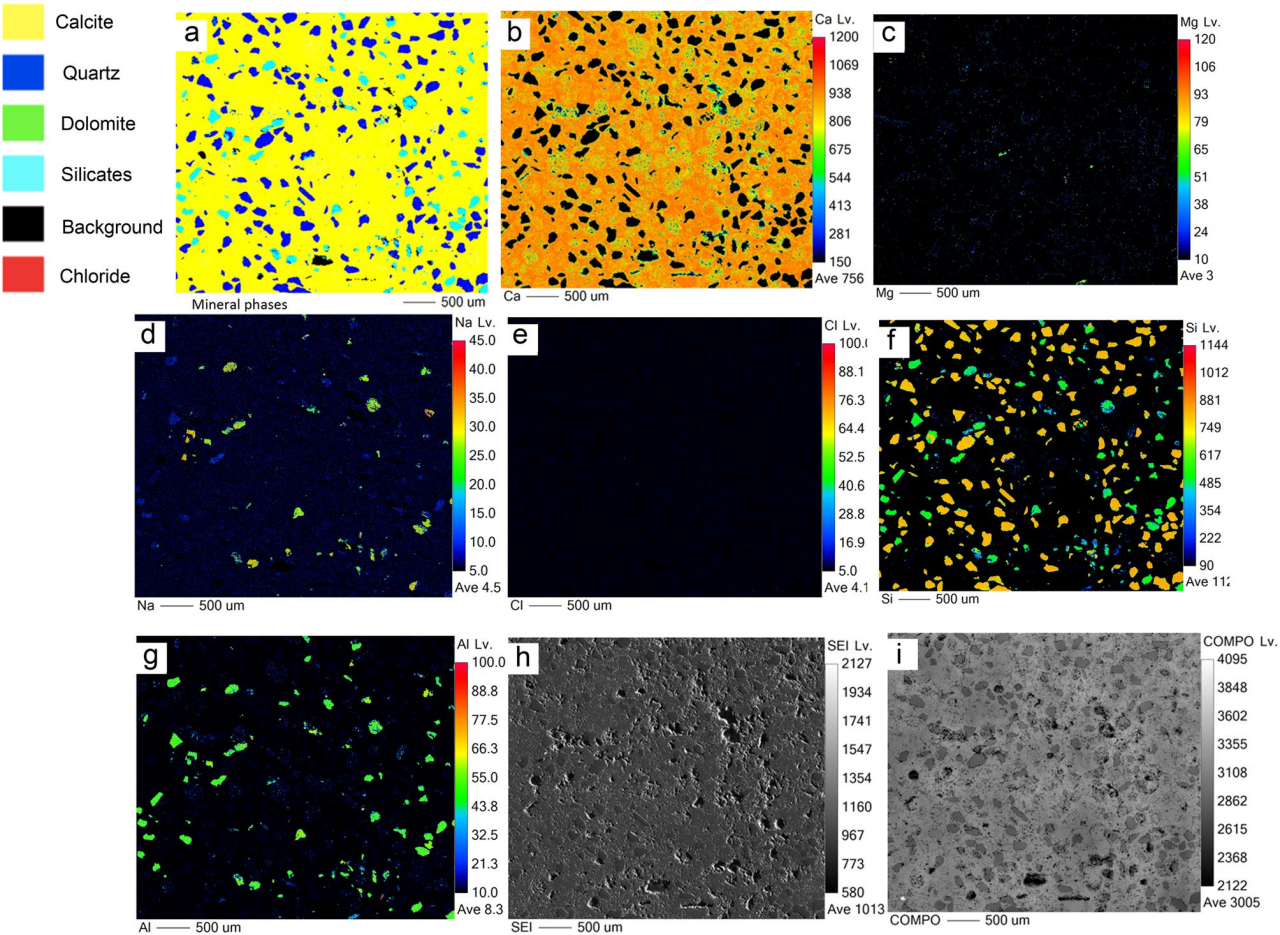
EPMA results of MV1 showing: (**a**) mineralogic maps, elemental maps of (**b**) Ca, (**c**) Mg, (**d**) Na, (**e**) Cl, (**f**) Si, and (**g**) Al, (**h**) secondary electron image, and (**i**) backscattered image. Dolomite and traces of salt are absent with textural homogenous matrix.

The details of mineral abundance in the rocks are analysed from EPMA data (Table 1) by calculating the normalized area to percentage from the compositional mineral phase maps. Figure 5 (MV1), Fig. 6 (MV2), and Fig. 7 (MV3) display the compositional mineral phase maps in panel ‘a’ and the elemental density maps of Ca, Mg, Na, Cl, Si, and Al in panels ‘b, c, d, e, f, and g’, respectively. The distribution of mineral phases is mapped based on the distribution of the constituent elements for each mineral phase. A noteworthy observation is the close relation between dolomite and chloride salt (NaCl) in the analysed samples. Dolomite and NaCl abundances are 22.91% and 2.51% in MV1, respectively, and 42.96% and 13.30% in MV2, respectively, while insignificant dolomite (1.05%) in MV3 corresponds to the absence of NaCl. It is important to note that the trivial dolomite revealed by EPMA in MV3 is not taken into account as EPMA calculates phase minerals based on elemental composition but not unit cell dimensions as XRD does. Thus, MV3 is reported as dolomite absent hereafter.

**Table 1:** EPMA results of mineral phase area % in MV1, MV2, and MV3.

Mineral phase	Sample		
	MV1	MV2	MV3
	Normalized area to 100%		
Calcite	54.73	36.58	83.60
Dolomite	22.91	42.96	1.05
Quartz	12.02	4.26	10.23
Silicates	7.84	2.89	5.12
Chloride salt	2.51	13.30	ND

In MV1, microcrystalline dolomite forms a mesh-like fabric in the calcite matrix (Fig. 5a) while dolomite in MV2 exhibits replacive cement of the pre-existing calcite matrix with clear delineation (Fig. 6a). Dolomitized fossils in MV2 are distinctive from Mg mapping (Fig. 6c) and their structures are recognizable in Na and Cl mapping (Fig. 6d,e). Interestingly, the mapping of Mg, Na, and Cl in MV2 are clearly superimposed. In MV3, the intensity of Mg (Fig. 7c) is very low in comparison with MV1 and MV2.

**Figure 8:**
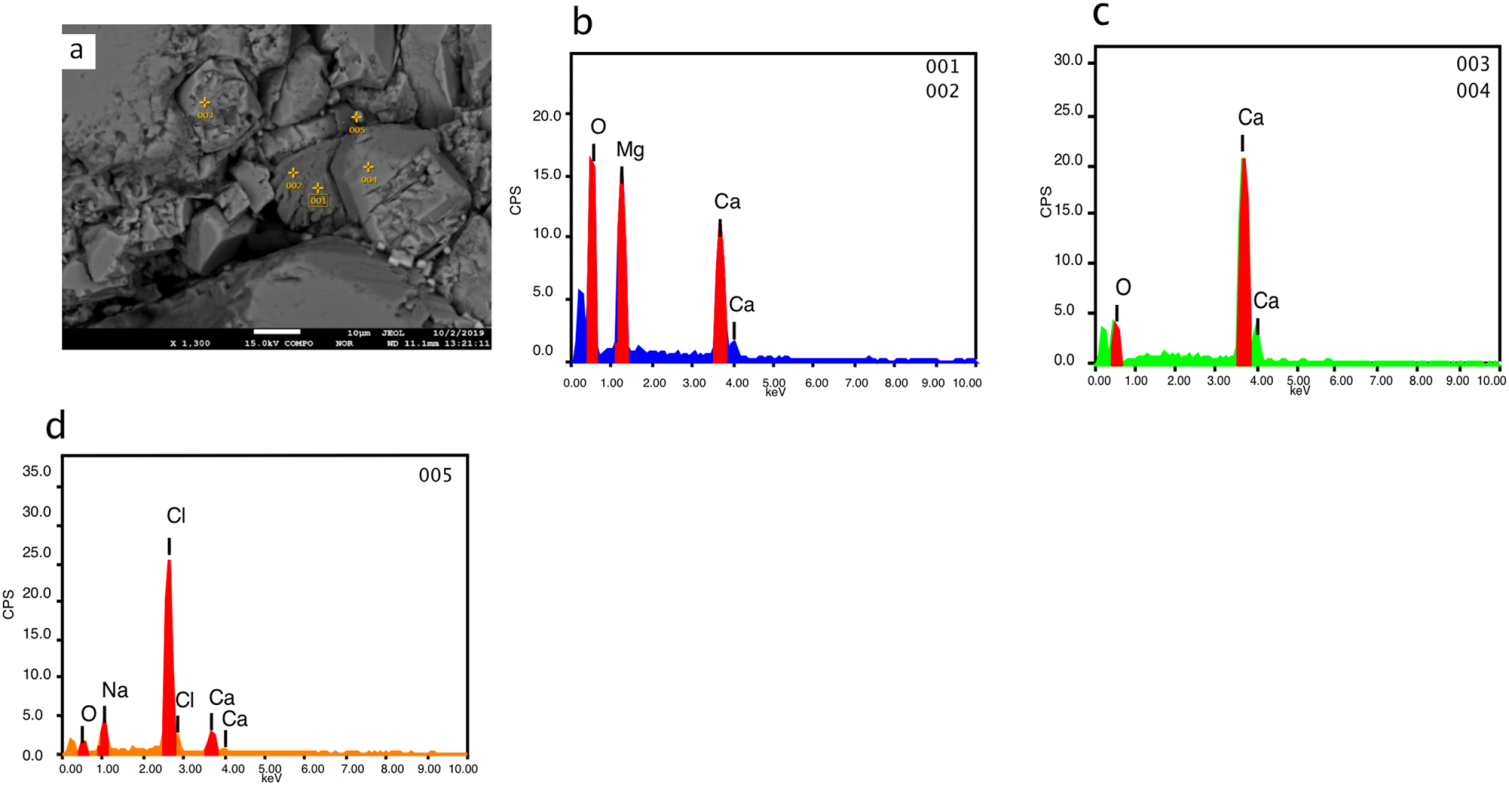
EPMA single point analyses of MV1 sample. (**a**) backscattered electron image of rhombohedral dolomite engrained within calcite matrix. The EDS chemical analysis shows the elemental composition of: (**b**) dolomite (points 001 and 002), (**c**) calcite (points 003 and 004), and (**d**) halite (point 005).

**Figure 9:**
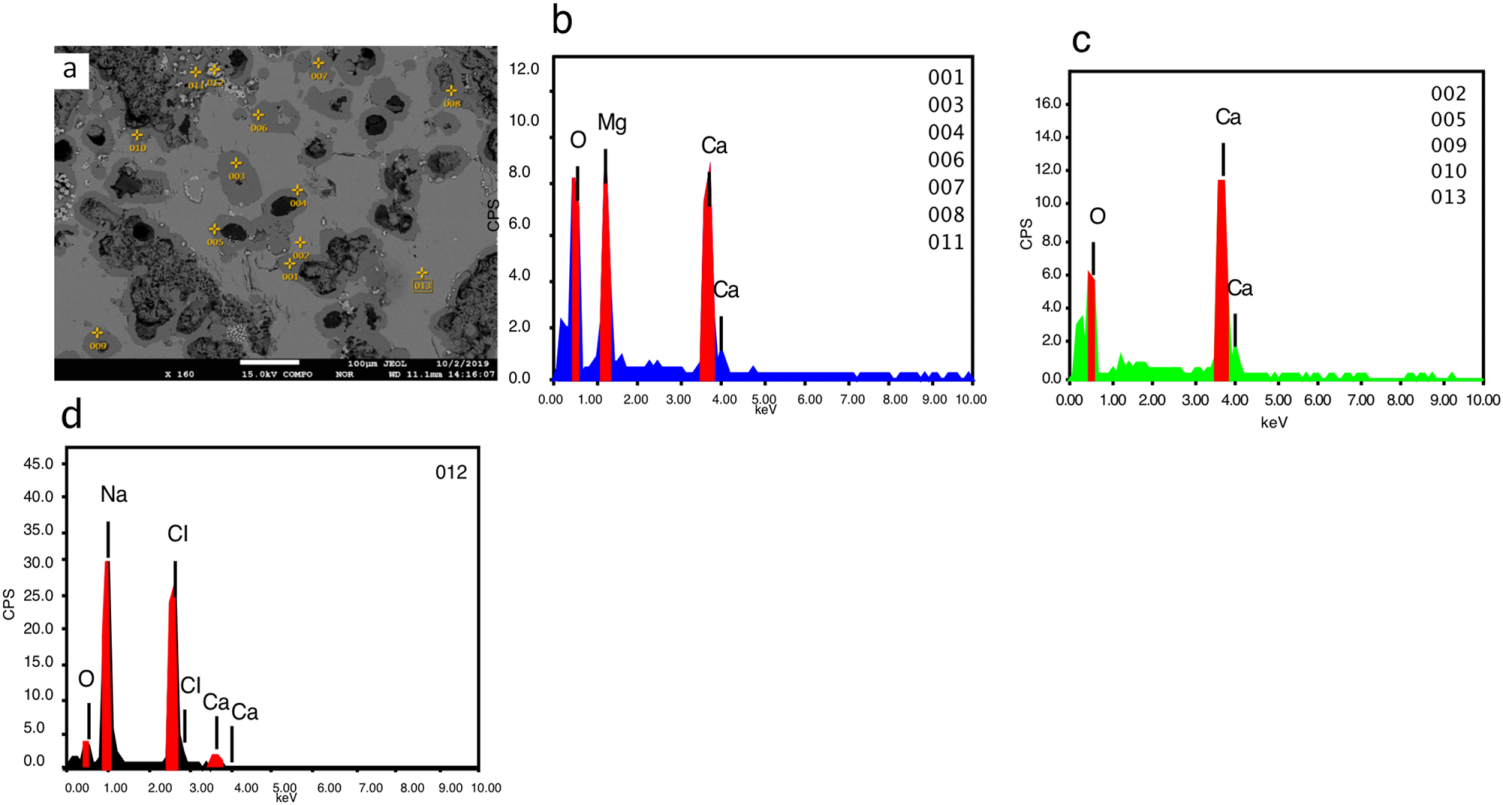
EPMA single point analyses of MV2 sample. (**a**) backscattered electron image of spheroidal dolomite with hollows suggesting channels for methane seepage. The EDS chemical analysis shows the elemental composition of: (**b**) dolomite (points 001, 003, 004, 006, 007, 008, and 011), (**c**) calcite (points 002, 005, 009, 010, and 013), and (**d**) halite (point 012).

**Figure 10:**
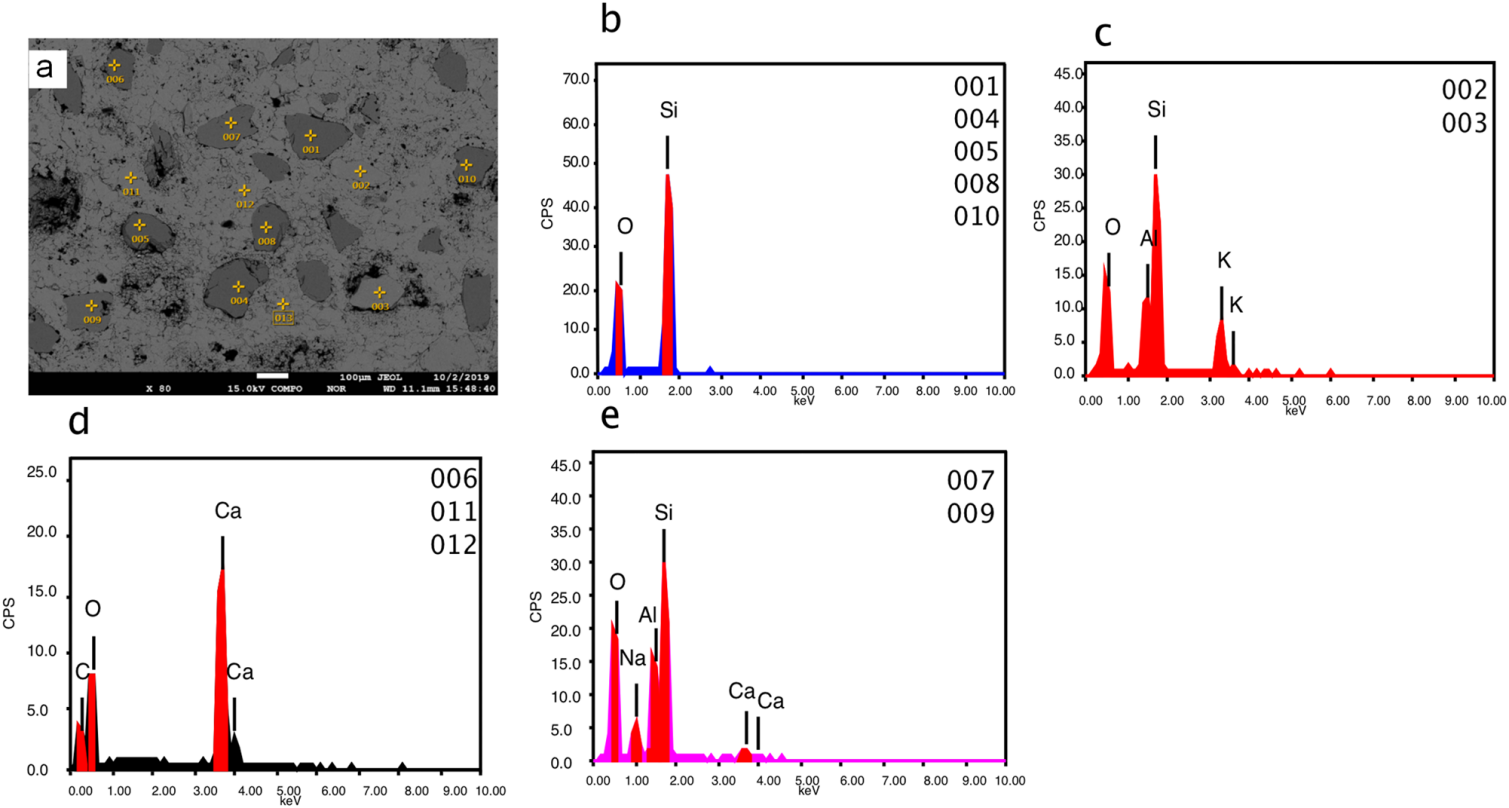
EPMA single point analyses of MV3 sample. (**a**) backscattered electron image of the calcitic matrix with clastic grains. The EDS chemical analysis shows the elemental composition of: (**b**) Quartz (points 001, 004, 005, 008, 010, 008), (**c**) silicates- K feldspar (points 002 and 003), (**d**) calcite (points 006, 011, 012), and (**e**) silicates- plagioclase (points 007 and 009). Neither dolomite nor salt traces were perceived.

Single point analyses with EDS in MV1 (Fig. 8) show an example of microcrystalline dolomite within calcite, large spheroidal dolomite replacing calcite in MV2 (Fig. 9), and dolomite absence in MV3 (Fig. 10). Dolomite stoichiometry in MV1 and MV2 were determined from single point analyses where Ca, Mg, Mn, and Fe of dolomite were quantified and compared to standard dolomite (Table 2). All analysed single points of dolomite show minor calcium enrichment in agreement with the XRD results (Fig. 4) with traces of Mn and Fe.

**Table 2:** Results of dolomite stoichiometry inferred from single point analyses from EPMA data. Elemental compositions are in mole% calibrated to standard dolomite. Traces of Fe and Mn are constituents of dolomite.

Point	Comment	MgO(mole%)	CaO(mole%)	FeO(mole%)	MnO(mole%)	CO_3_(mole%)	Mg/Ca
1	Dolomite standard	25.34	25.24	0.00	0.00	49.40	1.00
2	MV1-dol-1	22.60	25.90	0.27	0.02	51.25	0.87
3	MV1-dol-2	21.78	26.49	0.17	0.00	51.50	0.82
4	MV1-dol-3	23.71	26.20	0.17	0.02	49.85	0.91
5	MV2-dol-1	23.65	26.28	0.21	0.01	49.77	0.90
6	MV2-dol-2	23.23	26.78	0.18	0.02	49.70	0.87
7	MV2-dol-3	22.95	26.93	0.16	0.04	49.72	0.85

**Figure 11:**
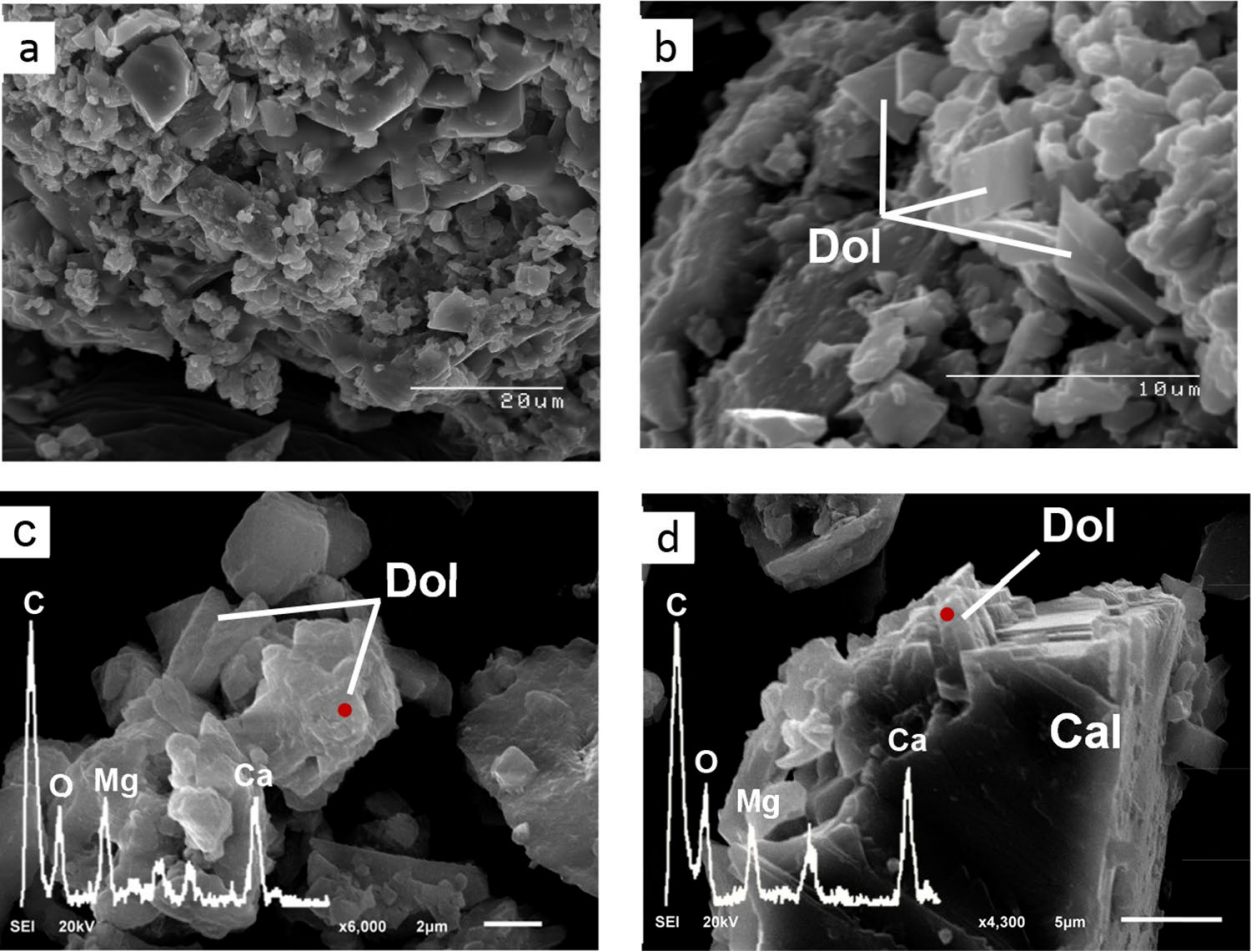
Scanning electron images of authigenic carbonate. (**a**) carbonate matrix represents microcrystals of rhombic dolomite. (**b**) rhombohedral dolomite with soft faces and well-defined edges. (**c**) Dolomite crystals with rough faces. (**d**) Intergrowth of rhombohedral dolomite and calcite showing dolomite precipitation at the expense of calcite dissolution. EDS spectra of single point analysis (**c**) and (**d**) are of the red points. Peaks of Os, Na, Cl, and Si in the spectra were not labeled.

The electron photomicrograph images with EDS analysis show planar texture of euhedral-subhedral microcrystal dolomite rhombs with size ranging from 2 to 5 μm. Dolomite rhombs are generally characterized by well-developed crystal faces and sharp boundaries; some rhombs demonstrate less distinctive characteristics. The growth of dolomite at the expense of calcite is noted from dolomite crystallization on a partially dissolved calcite (Fig. 11).

### Petrographic characterization

**Figure 12:**
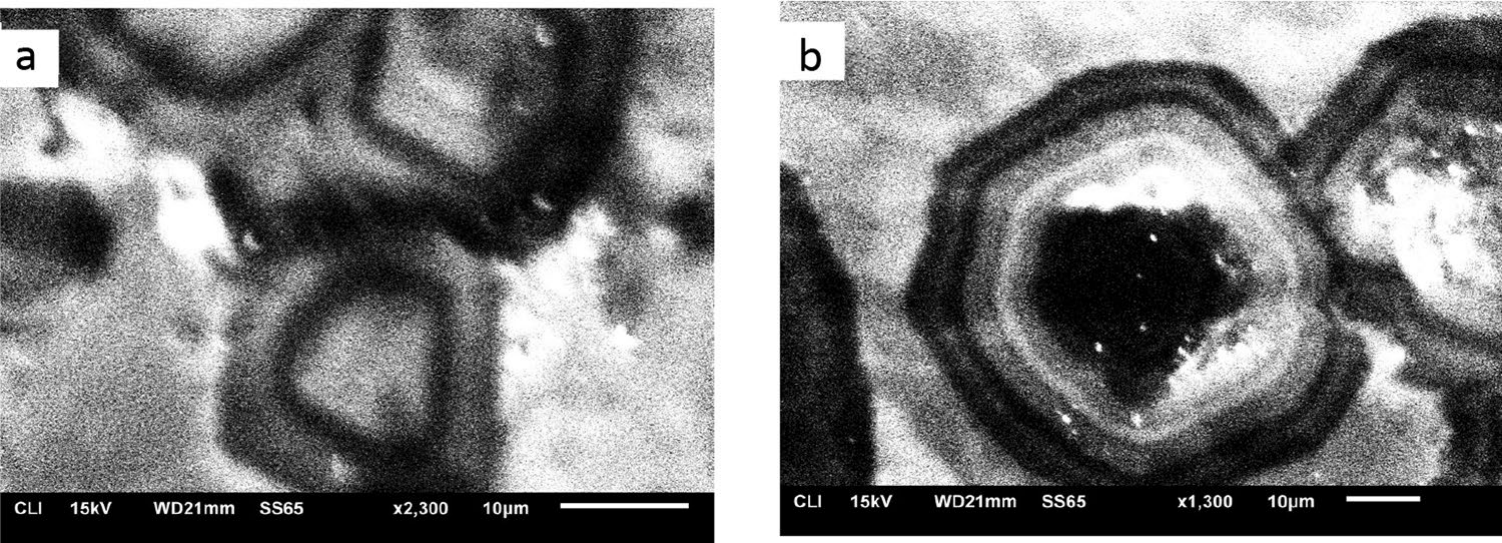
CL images (**a**) rounded spherulitic grains of dolomite. (**b**) zonation layers of dolomite with a hollow in the center of the grain. Dolomite zones show visible discontinuities where each zone is distinctive from the adjacent zones.

The fabric of spheroidal dolomite obtained via cathodoluminescence microscopy reveals radial concentric fabric with alternating layers of bright and dark luminescence (Fig. 12). Dolomite radial zonation shows visible discontinuities making each zone distinctive from the adjacent zone.

### Concentration of rare earth elements and trace elements

**Figure 13:**
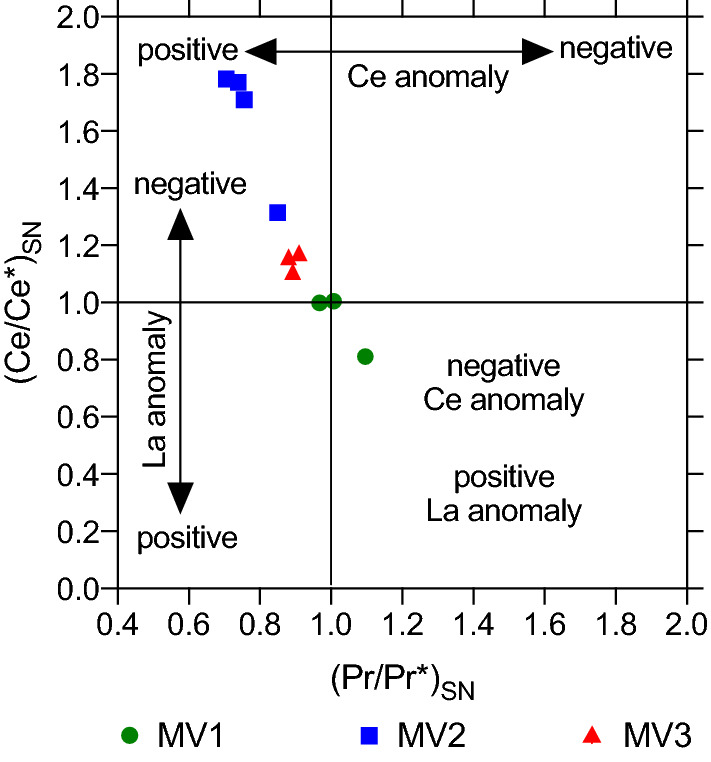
Relation between Ce/Ce* and Pr/Pr*in the Miocene mud volcano carbonates. MV2 shows the highest positive Ce anomaly while MV3 spots cluster at less intensive positive Ce. Two of MV1 spots show no anomalies and one indicate a negative Ce and positive La anomalies. This approach was described by (Bau and Dulski.,1996).

**Figure 14:**
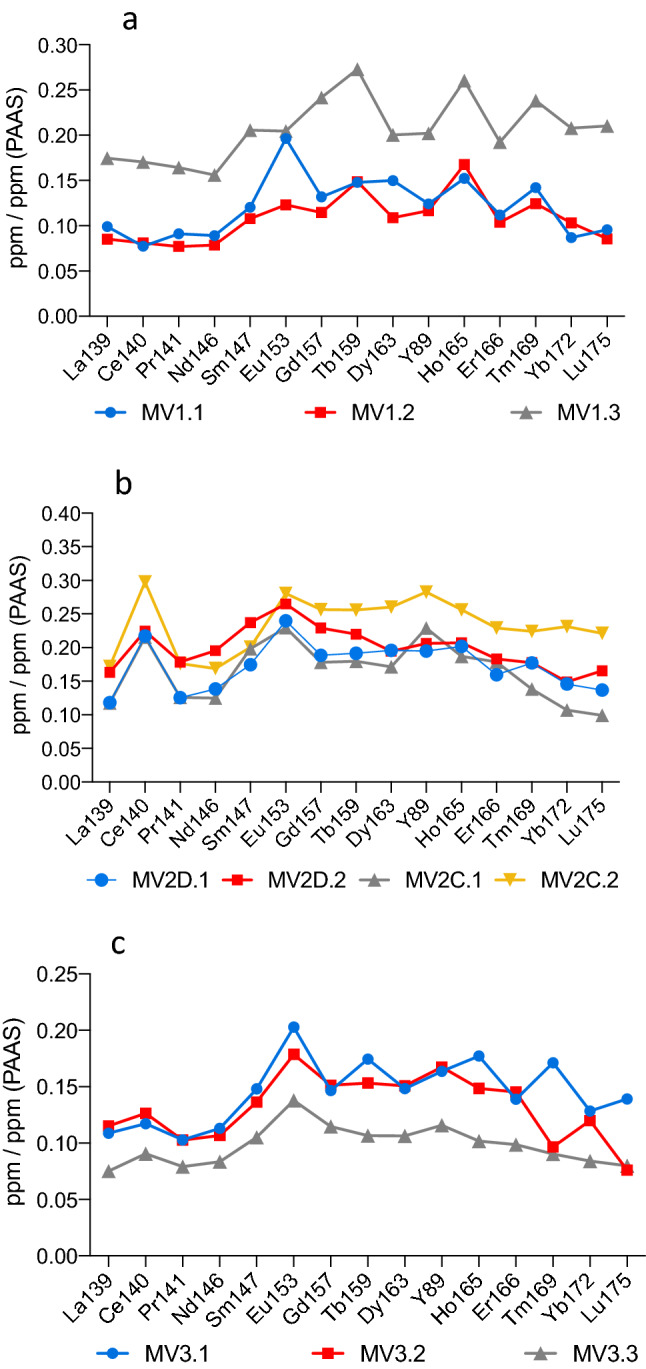
PAAS-normalized REY patterns for sub-samples of MV1 (**a**), MV2 (**b**), and MV3 (**c**). The results of MV1 represent the calcite matrix with microcrystalline dolomite, no differentiation between calcite and dolomite. MV2 shows distinctive results of calcite and dolomite separately and MV3 results represent the calcite matrix.

The concentrations of Rare Earth Elements and Yttrium (REY), Th, and U are listed in Table 3 with anomalies of PAAS-normalized (Ce/Ce*)sn, (Eu/Eu*)sn, and (Pr/Pr*)sn. Figure 13 shows the reducing conditions based on the results of (Ce/Ce*)sn and (Pr/Pr*)sn^45^. The PAAS-normalized REY patterns have shown different trends among the investigated rocks. Figure 14a denotes the results of three sub-samples from the MV1 rock with no discrepancy between dolomite and calcite. All sub-samples exhibit (Ce/Ce*)sn in the range between 0.8 to 1.2. MV1.1 indicates a slight positive La anomaly, negative Ce anomaly, and strong positive Eu anomaly. Overall, the patterns of MV1.2 and MV1.3 have ambiguous spikes compared to PAAS-normalized seawater signatures. Figure 14b represents MV2 results of calcite and spheroidal dolomite separately where sub-samples MV2D.1 and MV2D.2 represent dolomite while MV2C.1 and MV2C.2 represent calcite. The trends of dolomite and calcite patterns show a significant similarity except for Y anomalies since Y and Ho are decoupled in calcite patterns. Both dolomite and calcite show negative La and positive Eu anomalies. Ce anomalies of all MV2 sub-samples are positive (Ce/Ce*)sn > 1.2. Figure 14c displays patterns of calcite sub-samples in MV3 that show negative La anomalies, positive Ce anomalies (Ce/Ce*)sn ranging between 0.8 to 1.2, and strong positive Eu anomalies. MV3.1 and MV3.2 have spikes in heavy rare earth elements (Er, Tm, Yb, and Lu). The mean concentration of U in MV1, MV2, and MV3 is 1.36, 2.39, and 0.30 ppm, respectively, where the average Th/U ratio is 1.26, 0.6, and 1.1, respectively.

**Table 3:** Concentrations (ppm) of rare earth elements, Yttrium, Thorium and Uranium in the sub-samples of carbonate rocks from Miocene-age mud volcano outcrop. Y is inserted between Dy and Ho due to the chemical similarity^97^. Anomalies are normalized to PAAS^42^.

Element	MV1.1	MV1.2	MV1.3	MV2D.1	MV2D.2	MV2C.1	MV2C.2	MV3.1	MV3.2	MV3.3
La139	3.79	3.25	6.67	4.52	6.23	4.49	6.57	4.15	4.40	2.87
Ce140	6.15	6.45	13.56	17.29	17.88	17.15	23.69	9.33	10.05	7.21
Pr141	0.81	0.68	1.45	1.11	1.58	1.11	1.56	0.91	0.91	0.70
Nd146	3.02	2.67	5.29	4.69	6.63	4.23	5.72	3.83	3.62	2.83
Sm147	0.67	0.60	1.14	0.97	1.32	1.10	1.11	0.82	0.76	0.58
Eu153	0.21	0.13	0.22	0.26	0.29	0.25	0.30	0.22	0.19	0.15
Gd157	0.62	0.53	1.13	0.88	1.07	0.83	1.20	0.68	0.71	0.53
Tb159	0.11	0.12	0.21	0.15	0.17	0.14	0.20	0.14	0.12	0.08
Dy163	0.70	0.51	0.94	0.92	0.91	0.80	1.22	0.69	0.71	0.50
Y89	3.35	3.15	5.46	5.26	5.57	6.19	7.63	4.42	4.52	3.13
Ho165	0.15	0.17	0.26	0.20	0.21	0.19	0.25	0.18	0.15	0.10
Er166	0.32	0.30	0.55	0.46	0.52	0.51	0.65	0.40	0.41	0.28
Tm169	0.06	0.05	0.10	0.07	0.07	0.06	0.09	0.07	0.04	0.04
Yb172	0.25	0.29	0.59	0.41	0.42	0.30	0.65	0.36	0.34	0.24
Lu175	0.04	0.04	0.09	0.06	0.07	0.04	0.10	0.06	0.03	0.03
(Ce/Ce*)sn	0.81	1.00	1.00	1.78	1.31	1.77	1.71	1.11	1.16	1.17
(Eu/Eu*)sn	1.56	1.11	0.92	1.32	1.14	1.22	1.23	1.38	1.24	1.25
(Pr/Pr*)sn	0.89	0.88	0.91	0.71	0.85	0.74	0.76	1.10	0.97	1.01
Th232	1.25	1.26	2.47	1.35	1.53	0.70	1.96	0.30	0.39	0.25
U238	1.08	0.85	2.17	1.85	2.04	2.43	3.24	0.31	0.37	0.20
Th/U	1.16	1.48	1.14	0.73	0.75	0.29	0.61	0.97	1.05	1.27

## Discussion

### Dolomite related to hydrocarbon seepage and hypersaline seawater

The seepage of natural gas and oil can occur in different geological environments including active continental margins, passive continental shelves, open seas, and mud volcanoes^46^. Mud volcanoes belong to cold seep systems where the discharged fluids are transformed to authigenic carbonates because of microbial oxidation of methane and other hydrocarbons^47^. Dolomite formation in marine coastal hydrocarbon seeping environments is well-documented in the literature from different geographic locations including Barbados Accretionary Prism^48^, northern South China Sea^49^, Gulf of Mexico^50^, Eastern Mediterranean Sea^51^, south east Caspian Basin^52^, and Kuroshima Knoll in Japan^53^.

Our research on the mud volcano outcrop in north Kuwait shows the abundance of dolomite in the burrows and bioturbated vents from the marine zones where dolomite formation in these settings is primarily related to anaerobic oxidation of methane coupled with sulphate reduction^54^. In a Miocene mud volcano in Italy, dolomite cement precipitated around a channelized flux of methane-charged fluids forming cylindrical concretions where anaerobic oxidation of methane resulted in increasing alkalinity; consequently precipitating dolomite^55^. Cross sections of these dolomitized cylindrical concretions have similar features to the spheroidal dolomite found within MV2 sample in this study suggesting that the hollow cores in MV2 are probably the channels of methane emissions (Fig. 9a). Furthermore, spheroidal dolomite in MV2 has similar structural features of spheroids of isopachous chains found in calcite spar from a Miocene methane seep in Italy, which were assumed to be anaerobic microbial consortia responsible for methane oxidation^56^. Although important, hydrocarbon seepage does not explain the absence of dolomite in the continental zone of our studied mud volcano outcrop.

An important factor for dolomite formation in hydrocarbon seepage environments is the fluid of hypersaline seawater. Dolomite formed in areas of marine hydrocarbon seepage where sub-surface methane-charged fluids penetrated upwards thereby mixing with the shallow seawater in the anaerobic sediment where methane was oxidized by sulphate^53^. This finding may substantiate the preferential dolomitization of authigenic carbonates in the marine zones of our study area which is related to the evaporitic sabkha. The evaporated seawater in the sabkha contributes to high Mg/Ca ratio^17^ and is known to trigger dolomite formation^57^. Our results show clear evidence that the dolomite-comprising burrows and bioturbated MV1 and MV2 samples are from marine zones which is indicated by halite contents in XRD analyses (Figs. 3, 4) and foraminifera (Fig. 6e). Also, mineral phase abundance obtained from EPMA (Table 1) for the rocks demonstrate a close relationship between dolomite and NaCl. MV1 and MV2 constitute a considerable amount of dolomite and NaCl, while MV3 lacks dolomite and NaCl. Furthermore, the precipitation of megacrystic gypsum within MV1 zone is a reliable sign of high evaporation^58^. The microcrystalline dolomite in MV1 exhibits a mesh-like fabric represented by Mg mapping (Fig. 5c) possibly indicating a dissolution of susceptible dolomite^41^. Interestingly, a nanoscale *in-situ* observation using atomic force microscopy (AFM) has shown that the precipitation of gypsum is coupled with dolomite dissolution^59^. The absence of fossils in MV1 can be related to dolomitization in the upper tidal flat environment^60^. MV1 is possibly a re-worked carbonate material from the vents during sediment extrusion thereby abrading pre-existing earlier dolomite of spheroidal nature. In MV2, the superimposed distribution of Mg, Na, and Cl (Fig. 6c, d, e, respectively) suggest an interdependent relationship between dolomitization and hypersalinity. Indeed, high salinity provides the necessary ions for dolomite saturation as it has been previously demonstrated^6,12^. High salinity increases fluid ionic strength which facilitates the dehydration of Mg from Mg-H_2_O complexes via the formation of hydration shells around Na and Cl ions^61^. Consequently, the Mg-H_2_O complexes become less stable and Mg becomes more available for dolomite crystal formation and growth^61^. Thus, the co-existence of high salt concentration, marine fossils, and dolomite is an indicator for the involvement of hypersaline seawater in dolomitization. On the other hand, the absence of NaCl has been found in the continental MV3 sample together with the low intensity of Mg in MV3 (Fig. 7c).

### The impact of bioturbation on dolomite formation and diagenesis

Bioturbation refers to displacement within sediments introduced by organisms while ichnofabric is the recorded texture and structure in the sediment from the bioturbation activity ^62^. Dolomite in this study was found in the bioturbated sediment and the burrows. Commonly, dolomite in burrows is associated with reducing conditions^63^, sulphate-reducing bacteria^25,64–66^, and marine-derived organic constituents^67^, but this association with seep carbonate in evaporitic rocks is new.

The bioturbation structures in authigenic carbonates of the investigated mud volcano in this study provides evidence that the marine zones are the host of the dolomitized crustacean burrows whose origins were previously described as perplexing^25^. Remarkably, dolomite stoichiometry in our samples (Table 2) is almost identical to the composition (Ca_52_Mg_48_ and Ca_57_Mg_43_) of selectively dolomitized crustacean burrows described by Gunatilaka et al*.* (1987)^25^.

The selectively dolomitized burrows result from the effect of crustaceans on modifying the physical and chemical properties of the sediment where burrows act as a conduit for the sediment–water interface^68^. These conduits provide channels for the flow of fluids from sub-surface hydrocarbon reservoirs^32^ and supply Mg^2+^ to the burrows from interstitial marine sources^68^. In the microenvironment of the burrows, dolomite is generally prompted by a consortium of methane-oxidizing archaea and sulphate-reducing bacteria that mediate the sulphate-driven anaerobic oxidation of methane (SD-AOM), and produce bicarbonate and dissolved sulphide^36^. Moreover, sulphate reduction in the burrows can release Mg^2+^ ions from neutral ion pairs which raises the concentration of Mg^2+^ for the dolomite-filled burrows^63^.

The patchy and mosaic dolomite in MV1 and MV2, respectively, created diagenetic textural heterogeneities depicted by comparing the mapping of dolomite (Figs. 5a, 6a) with the corresponding backscattered images (Figs. 5i, 6i). The textural heterogeneities of the calcite matrix contemporaneous to dolomitization is an indication of burrow-associated dolomite^69^. Additionally, the dolomitized fossils of fragmental shells and probably foraminifera (Fig. 6e) is direct evidence of diagenesis^70^ where the microenvironment of burrows provides the favourable geochemical conditions for dolomitization^68^, and microfossils seem to act as a *loci* for dolomitization. The preserved annular radial pattern in MV3 rock (Fig. 2f) sampled from the continental zone implies no diagenetic features and exhibits homogeneous texture (Figs. 7h, i). Not surprisingly then, that the diagenetic fabrics are indicative for dolomitizing fluids that seeped through burrow networks^68^, which explains the genesis of dolomite in the marine mud vents of Al-Subiya where prolific *Ophiomorpha* and *Thalassinoides* were described^28^.

The preserved radial concentric Cathodoluminescence (CL) zonation of dolomite in MV2 (Fig. 12) can be recognized in textural association with multiple episodes of meteoric diagenesis related historically to transgression/regression^71^. Furthermore, dolomite formation in continental evaporitic environment can have a dual source of mixed meteoric/seawater such as in coastal lakes and lagoons of the Coorong region of south Australia^72^. The dull and luminescent zonation is controlled by varying concentrations of Mn and Fe^73^ which describe the geochemical changes throughout dolomite diagenesis^74^. An unconfined aquifer would be oxygenated via vadose zone recharge resulting in Fe and Mn oxidation and dull luminescence. In contrast, the luminescence becomes bright when no recharge from vadose zone reaches the confined aquifer^71^. Nevertheless, we believe that the oxygenation of the aquifer in our case cannot be solely related to vadose recharge as methane seepage is part of the studied environmental setting. As such, methane pulsive seepage can also affect the pattern of CL zonation by changing the redox state of the environment and consequently the availability of Mn and Fe^75^.

### Geochemical signatures of authigenic carbonates

The distinct distribution of PAAS-normalized seawater Rare Earth Elements and Yttrium (REY) can be authentically preserved in carbonate sediment and rocks^76^. This section provides evidence of the geochemical conditions governing the process of carbonate formation in the studied mud volcano. Rare Earth Elements and Yttrium (REY) have been considered reliable proxies for understanding the geochemistry of marine environments^77,78^.

A fundamental geochemical signature of the reducing environment is Ce anomaly; a proxy used for constructing oxic, suboxic, and anoxic conditions of ancient marine environments^79,80^. Under oxidizing conditions, Ce^3+^ is oxidized to less soluble Ce^4+^ scavenged by suspended particles and settling through the water column^77^. Therefore, the carbonates that have precipitated in equilibrium with seawater commonly demonstrate removed Ce and thus negative Ce anomalies^81^. The reducing environmental conditions were determined from the plot of Ce and La anomalies calculated from Pr/Pr* = Pr_sn_/(0.5Ce_sn_ + 0.5Nd_sn_) and Ce/Ce* = Ce_sn_/(0.5Pr_sn_ + 0.5La_sn_)^82^ (Fig. 13). MV1 and MV3 sub-samples indicate no true Ce anomalies as (Ce/Ce*)sn values lie between 0.8 and 1.2, suggesting carbonate formation under suboxic to anoxic conditions (Hu et al., 2014). On the other hand, MV2 sub-samples have shown true positive Ce anomalies (> 1.2) indicating dolomite and calcite were precipitated under strictly anaerobic reducing conditions (Hu et al., 2014) (Fig. 13). Moreover, a positive Ce anomaly can be due to intensive alkaline water conditions^83^ since highly alkaline water can transform insoluble Ce to soluble complexes thereby resulting in positive Ce anomaly in carbonate sediments^84^. The implications of anoxic conditions from Ce anomalies may indicate a presence of methane related to hydrocarbon seepage ^85^ where the pockmarks found in the mud volcano outcrop (Fig. 2i) are indicative of methane bubbles^86^. Indeed, the formation of authigenic carbonates including dolomite related to methane seepage under anoxic conditions is well-documented in the literature^34,53,68^.

The patterns of REY trends in MV1 sub-samples (Fig. 14a) are inconsistent with the presence of inexplicable spikes; possibly due to the alteration of carbonate rock composition as a result of diagenesis^74^, admixture of dust and detrital organic matter^87^, or terrigenous contamination^88^. These patterns of MV1 sub-samples are referred to as the heterogenous matrix with no discrepancy between dolomite and calcite. The patterns of dolomite and calcite sub-samples in MV2 (Fig. 14b) show strong Eu anomalies which may denote the input of hydrothermal fluids to seawater^82,89^. The implication of the positive Eu anomalies is supported by the finding of upward movement of hydrothermal fluids observed nearby our study site at Bahrah field which is linked to seal breach in subsurface carbonate petroleum systems^90^. Additionally, the positive Eu anomaly indicates sulphate reduction to sulphide^91^ which is compatible to the metabolic pathway of sulphate-reducing bacteria contributing to anaerobic methane oxidation^53^. Interestingly, the patterns of MV2D.1 and MV2D.2 of dolomite are identical to that of Congo Fan seep carbonate that formed under sulphidic conditions and significant anaerobic oxidation of methane^50^. The patterns of calcite in sub-samples MV2C.1 and MV2C.2 show positive Y anomalies that are absent in dolomite sub-samples MV2D.1 and MV2.D2. The positive Y anomalies are a signature of seawater^76^ suggesting that the absence of Y anomaly in dolomite can be associated with subsurface ejecting fluids. The noticeable strong positive Eu signature in MV3 sub-samples pattern of calcite (Fig. 14c) indicates the involvement of hydrothermal fluids. The trend of MV3.1 from Gd to Lu possesses no seawater signature while pattern of MV3.2 and MV3.3 from Gd to Lu have unexplained spikes which may be due to terrigenous contamination.

An additional important proxy for reducing conditions is the Th/U ratio where lower ratios reveal more reducing conditions^92^. The average Th/U for MV1, MV2, and MV3 is 1.26, 0.59, and 1.09, respectively, where the minimum ratio in MV2 shows the most reducing conditions matching the results of real positive Ce anomalies (Fig. 13).

**Figure 15:**
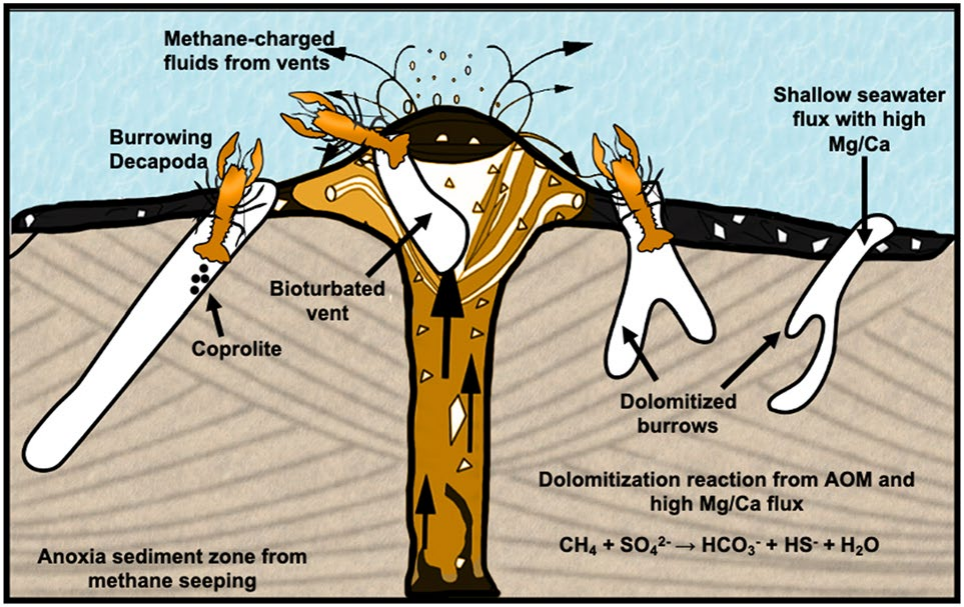
A schematic diagram adapted with permission from Hyžný et al*.* (2018) describing dolomite formation in bioturbated vents and burrows from the coastal mud volcano. The scheme was modified using Adobe Photoshop version 22.0.1 (https://www.adobe.com/).

Our model of dolomite formation in the early-middle Miocene coastal mud volcano outcrop in north Kuwait is depicted in Fig. 15. In summary, dolomite in the studied early-middle Miocene mud volcano outcrop is associated with the activity of decapod crustaceans in the marine zones. The activity of burrowing crustaceans is well documented in seep carbonates worldwide ^93,94^ where the burrows serve as channels of fluid for vertical flow within the sediment ^69^. Burrowing crustaceans can thrive in environmental stresses of high salinity and low oxygen levels exemplified by a sabkha environment in the Mississippian Debolt Formation located in Northwestern Alberta, Canada. These conditions have created dolomitizing bioturbated fabrics and the burrows themselves are dolomitized in the presence of microbial sulphate-reduction^95^. The ability of some intertidal burrowing crustaceans such as *Thalassinideans* to tolerate anoxia for 50–60 h ^96^ can explain the presence of burrows in anoxic sediment zones. In methane rich environments, the open crustacean dwelling structures (*Thalassinoides*) act as a seepage discharge that preferentially focuses saturated water and methane-charged fluids, resulting in burrow cementation mediated by AOM ^94^.

## Conclusion

We have re-evaluated the genesis of dolomite in north Kuwait by investigating recently discovered early-middle Miocene mud volcano outcrops within Al-Subiya sabkha. Our results suggest that interdependent events of hydrocarbon seepage, hypersaline seawater, and bioturbation led to dolomite formation in the coastal mud volcano. Our findings show that burrows can provide a preferential geochemical microenvironment for dolomite genesis within marine mud volcanoes due to the mixing of ascending methane-charged fluids with overlying seawater, in the bioirrigated sediment. The involvement of methane in dolomite formation is implied from the positive Ce anomaly that reflects strictly anoxic reducing conditions. Furthermore, the positive Eu anomaly of dolomite suggests that sulphidic conditions is likely coupled with anaerobic oxidation of methane. The importance of high salinity for dolomitization was supported by the presence of dolomite and NaCl in the marine zones and the distorted rock fabric is indicative of diagenesis. These results propose a new diagenetic model of dolomitization in the Arabian Gulf coastlines where most other diagenetic models were reported to be consequences of sabkha flood recharge/reflux, and concentrated seawater.
